# Host-Pathogen Interactions: What the EHEC Are We Learning from Host Genome-Wide Screens?

**DOI:** 10.1128/mBio.01837-18

**Published:** 2018-10-09

**Authors:** Jason P. Lynch, Cammie F. Lesser

**Affiliations:** a Department of Medicine, Division of Infectious Diseases, Massachusetts General Hospital, Cambridge, Massachusetts, USA; b Department of Microbiology and Immunobiology, Harvard Medical School, Boston, Massachusetts, USA; c Broad Institute of MIT and Harvard, Cambridge, Massachusetts, USA

**Keywords:** EHEC, *Shigella*, *Vibrio parahaemolyticus*, CRISPR/Cas9 screen, type III secretion system

## Abstract

Several genome-wide screens have been conducted to identify host cell factors involved in the pathogenesis of bacterial pathogens whose virulence is dependent on type III secretion systems (T3SSs), nanomachines responsible for the translocation of proteins into host cells. In the most recent of these, Pacheco et al. (mBio 9:e01003-18, 2018, http://mbio.asm.org/content/9/3/e01003-18.full) screened a genome-wide CRISPR/Cas9 (clustered regularly interspaced short palindromic repeats with Cas9) knockout library for host proteins involved in the pathogenesis of enterohemorrhagic Escherichia coli (EHEC). Their study revealed an unrecognized link between EHEC’s two major virulence determinants (its T3SS and Shiga toxins). We discuss these findings in light of data from three other genome-wide screens. Each of these studies uncovered multiple host cell determinants, which curiously share little to no overlap but primarily are involved in mediating early interactions between T3SSs and host cells. We therefore consider how each screen was performed, the advantages and disadvantages of each, and how follow-up studies might be designed to address these issues.

## Abstract (precis)

Several genome-wide screens have been conducted to identify host cell factors involved in the pathogenesis of bacterial pathogens whose virulence is dependent on type III secretion systems (T3SSs), nanomachines responsible for the translocation of proteins into host cells. In the most recent of these, Pacheco et al.

## COMMENTARY

Enterohemorrhagic Escherichia coli (EHEC) is responsible for outbreaks of bloody diarrhea. While usually self-limited, in 5 to 7% of cases, this gastrointestinal illness can progress to hemolytic-uremic syndrome (HUS), a clinical syndrome characterized by the development of a hemolytic anemia, thrombocytopenia (low platelet count), and acute kidney disease. HUS primarily affects children under the age of 5 years and can be life-threatening. Treatment options are limited to supportive measures, including hydration and, if needed, renal replacement therapy (i.e., dialysis).

The pathogenesis of EHEC is dependent on its type III secretion system (T3SS), a complex nanomachine, which acts to translocate proteins directly into the cytosol of mammalian cells. In addition, EHEC secretes one or more Shiga toxins that underlie the development of HUS. As the expression of some prophage-embedded Shiga toxins is induced by stress, antibiotics are counterindicated in the treatment of infected individuals. Interestingly, a recent study by Pacheco and colleagues, based on the findings of a genome-wide screen for host factors involved in pathogenesis, suggests a previously unrecognized link between these two important virulence determinants that can be exploited for the development of new therapeutic interventions for this important pathogen (1).

Genome-wide screens provide an unbiased approach to discover novel genes and pathways underlying biological processes and have been utilized for many decades to identify and characterize bacterial virulence factors. More recently, similar strategies have begun to identify the host cell determinants required for infection. Genome-wide screens for factors involved in mediating host-pathogen interactions were initially conducted using RNA interference (RNAi)-based technologies (2, 3). However, given concerns for issues with off-target effects from RNAi approaches, recent efforts have turned toward conducting CRISPR/Cas9 (clustered regularly interspaced short palindromic repeats with Cas9)-based studies with a variety of human cell lines (4, 5). This technology has proven to be particularly fruitful when screening for proteins required for the uptake of lethal toxins (i.e., Clostridium difficile toxin B and anthrax toxin [6, 7]). The strong selective pressure for cells resistant to toxin uptake enables the screening of pools of host cells, as opposed to arrayed libraries, saving in both cost and labor.

In order to conduct a genome-wide loss-of-function CRISPR/Cas9-based screen of a pool of intestinal epithelial HT-29 cells, Pacheco and colleagues first needed to identify infection conditions that promote significant host cell death. This is not a feature of infection with wild-type EHEC, but is observed with strains that lack EspZ, a type III secreted effector that negatively regulates the secretory activity of the EHEC T3SS (8, 9). Pacheco and colleagues cleverly chose this strain for their genome-wide screen. However, as their infection conditions only resulted in 80% cell death, to enrich their selection for loci whose loss confers resistance, they evaluated cells that survived four rounds of infection, repeating each after an ∼5-day outgrowth.

At the completion of their screen, Pacheco and colleagues identified thirteen loci whose absence conferred loss of susceptibility to infection with EHEC. Remarkably, six of these loci correspond to genes involved in the biosynthesis of sphingolipids, major components of the outer leaflet of the plasma membrane of eukaryotic cells. This observation was particularly surprising as Gb3 (globotriaosylceramide), a sphingolipid, serves as the cellular receptor that mediates the uptake of Shiga toxins into host cells. In order to confirm the results of the screen, as well as to generate a means to further investigate the role of sphingolipids in EHEC pathogenesis, the investigators generated HT-29 cell lines each of which no longer carries one of six of the loci identified in the screen, including four involved in sphingolipid biosynthesis and two of unknown function, which they later demonstrated to play a role in Gb3 biosynthesis. Notably, each of these strains demonstrated decreased levels of cell death when infected with a *ΔespZ ΔΔstx* EHEC mutant, a strain that lacks Shiga toxins. Thus, the Pacheco study strongly implicates a connection between host sphingolipids and the EHEC T3SS.

T3SSs are membrane-embedded nanomachines used by EHEC and other Gram-negative pathogens to translocate dozens of proteins, often referred to as effectors, into the cytosol of mammalian cells. The outermost structure of the T3SS, a complex of proteins referred to as the translocon, inserts into and forms a pore in the host cell membrane to complete the conduit that enables protein transport into host cells. Prior studies had demonstrated a role for lipid rafts in translocon insertion, at least in the context of the Shigella T3SS (10, 11). Interestingly, in the study by Pacheco and colleagues, the cell lines predicted to be impaired in sphingolipid metabolism each demonstrated markedly decreased levels of translocated Tir, a highly abundant EHEC effector. Similarly, consistent with decreased Tir translocation, the host cells were also impaired in the formation of actin pedestals that EHEC use to attach onto host cells, a Tir-dependent process. These observations strongly suggest a role for sphingolipids in the ability of the EHEC T3SS to translocate proteins into host cells.

Several additional genome-wide screens have been conducted for host cell factors involved in the pathogenesis of bacteria whose virulence is dependent on one or more T3SSs. For example, using the same HT-29 CRISPR/Cas9 library, Blondel and colleagues screened for loci whose absence confers resistance to cytotoxicity linked to each of the two T3SSs of Vibrio parahaemolyticus, T3SS1 and T3SS2 (12). These two screens identified additional host cell surface membrane modifications that result in decreased T3SS-linked cytotoxicity, but found no evidence for a role for sphingolipids. In the case of T3SS1, they discovered that loss of sulfation results in decreased V. parahaemolyticus adherence, thus, indirectly limiting T3SS1-mediated cytotoxicity. In the case of T3SS2, they found that loss of host cell glycan fucosylation inhibits membrane insertion of its translocon apparatus, limiting killing associated with this system.

In another study, Russo and colleagues conducted a genome-scale insertional mutagenesis screen of Hap-1 cells to identify loci involved in promoting an infection by Shigella flexneri (13). Among their 81 hits, which did not include proteins involved in sulfation, fucosylation, or sphingolipid biosynthesis, was vimentin, an intermediate filament. In follow-up studies, they determined that vimentin is required for the docking of the S. flexneri translocon apparatus onto host cells. They further demonstrated vimentin knockout cells experience markedly decreased translocation of not only effectors via the S. flexneri T3SS, but also closely related Salmonella enterica serovar Typhimurium SP1 T3SS and the more distally related Yersinia pseudotuberculosis Ysa T3SS. They did not investigate whether intermediate filaments play a role in regulating the activity of the EHEC or V. parahaemolyticus T3SSs.

Lastly, in a related genome-wide screen, Sheahan and Isberg directly screened for host cell proteins involved in the translocation of YopE, a Y. pseudotuberculosis effector, into cells. In this case, rather than screen for loci involved in preventing T3SS-linked cytotoxicity, they used a FRET (fluorescence resonance energy transfer)-based assay that directly monitors the activity of YopE, a Rho GTPase (14). They then sorted a pooled RNAi library of infected 293T cells for those that exhibited decreased YopE activity. From a screen of just 10,000 short hairpin RNAs (shRNAs), which covered ∼5,000 open reading frames (ORFs), the investigators obtained ∼300 hits. Follow-up work suggests that the majority of these hits, including those involved in regulating RhoA signaling, the formation of clathrin-coated pits, and receptors involved in cell signaling, including CCR5, the chemokine receptor involved in HIV host cell entry, all act to impair the ability of the Yersinia translocon apparatus to form pores in host cell membranes.

Collectively, four genome-wide screens have been conducted to identify host cell factors involved in mediating host-pathogen interactions. Curiously, little to no overlap in host cell resistance determinants has yet to be identified. This could potentially reflect inherent differences in (i) the ways that T3SSs interact with host cells, (ii) the cell types studied, each of which likely differs in its complement of expressed as well as essential proteins, and/or (iii) screen design. For example, by assessing their hits after multiple rounds of outgrowth postexposure to EHEC, Pacheco and colleagues potentially enriched for loci that provide protection against early T3SS and delayed Shiga toxin-mediated toxicity. While from a pathogenesis perspective, it would be interesting to see whether additional hits would be identified if the same library was screened with Shiga toxin-negative EHEC, their discovery of a role for glycolipids, and particularly Gb3, in mediating the activity of the EHEC T3SS, raises exciting new possibilities for the development of therapeutic interventions for this potentially deadly disease.

Notably, in each of the genome-wide screens described above, the investigators primarily identified host cell determinants involved in early steps in type III secretion, specifically the translocation of effectors into host cells. This is perhaps not too surprising, as each of these T3SSs delivers multiple and in some cases tens of effectors into host cells. These effectors target a variety of host cell proteins and processes, which together act to enable the pathogens to replicate and survive. Strains that lack individual effectors often do not exhibit major defects in essential steps in pathogenesis: thus, presumably loss of an individual host cell target will similarly not result in major changes, particularly when resistance to bacterium-triggered cytotoxicity is used as a readout. Future genome-wide screens seeking to identify host cell proteins involved in specific steps of bacterial pathogenesis will likely require additional approaches, such as cell-based, high-throughput, microscopy-based assays, which will grow increasingly accessible as more arrayed CRISPR/Cas9 libraries become available, or fluorescence-activated cell sorter (FACS)-based sorting assays. Thus, the true potential of genome-wide screens for the discovery of novel host determinants has likely not yet been fully realized.
